# A modified Stokes-Einstein equation for A*β* aggregation

**DOI:** 10.1186/1471-2105-12-S10-S13

**Published:** 2011-10-18

**Authors:** Srisairam Achuthan, Bong Jae Chung, Preetam Ghosh, Vijayaraghavan Rangachari, Ashwin Vaidya

**Affiliations:** 1 Neuroscience Center of Excellence, Louisiana State University Health Sciences Center, 2020 Gravier Street, Suite D, New Orleans, LA, 70112, USA; 2 Department of Mechanical Engineering, University of Pittsburgh, Pittsburgh, PA 15261, USA; 3 Department of Computer Science, Virginia Commonwealth University, Richmond, VA-23284, USA; 4 Department of Chemistry and Biochemistry, University of Southern Mississippi, 118 College Drive, Box No.5043, Hattiesburg, MS-39406, USA; 5 Department of Mathematical Sciences, Montclair State University, Montclair, NJ 07043, USA

## Abstract

**Background:**

In all amyloid diseases, protein aggregates have been implicated fully or partly, in the etiology of the disease. Due to their significance in human pathologies, there have been unprecedented efforts towards physiochemical understanding of aggregation and amyloid formation over the last two decades. An important relation from which hydrodynamic radii of the aggregate is routinely measured is the classic Stokes-Einstein equation. Here, we report a modification in the classical Stokes-Einstein equation using a mixture theory approach, in order to accommodate the changes in viscosity of the solvent due to the changes in solute size and shape, to implement a more realistic model for A*β* aggregation involved in Alzheimer’s disease. Specifically, we have focused on validating this model in protofibrill lateral association reactions along the aggregation pathway, which has been experimentally well characterized.

**Results:**

The modified Stokes-Einstein equation incorporates an effective viscosity for the mixture consisting of the macromolecules and solvent where the lateral association reaction occurs. This effective viscosity is modeled as a function of the volume fractions of the different species of molecules. The novelty of our model is that in addition to the volume fractions, it incorporates previously published reports on the dimensions of the protofibrils and their aggregates to formulate a more appropriate shape rather than mere spheres. The net result is that the diffusion coefficient which is inversely proportional to the viscosity of the system is now dependent on the concentration of the different molecules as well as their proper shapes. Comparison with experiments for variations in diffusion coefficients over time reveals very similar trends.

**Conclusions:**

We argue that the standard Stokes-Einstein’s equation is insufficient to understand the temporal variations in diffusion when trying to understand the aggregation behavior of A*β*42 proteins. Our modifications also involve inclusion of improved shape factors of molecules and more appropriate viscosities. The modification we are reporting is not only useful in A*β* aggregation but also will be important for accurate measurements in all protein aggregation systems.

## Introduction

Aberrant misfolding and aggregation of proteins have been implicated in over 40 different human pathologies including Alzheimer’s disease (AD), Parkinson’s disease, type 2 diabetes, transmissible spongiform encephalopathies (TSE) or Prion diseases, Huntington’s disease and Cruedfelt-Jacob disease (CJD). A biochemical commonality in these diseases is that the protein involved forms pathogenic aggregates, irrespective of whether the monomeric protein is in the misfolded or intrinsically disordered forms, that have a consensus structural moiety, commonly known as “amyloids”. In all amyloid diseases, such protein aggregates have been implicated fully or partly, in the etiology of the disease. Due to their significance in human pathologies, there have been unprecedented efforts towards physiochemical understanding of aggregation and amyloid formation over the last two decades. It is well understood that the process of aggregation towards amyloid fibrils from a monomeric state is a nucleation-dependant mechanism, analogous to crystal growth. In such a process, it is believed that there is a conformational change during the nucleation event followed by rapid aggregation to a state that forms large insoluble or soluble aggregates, which in turn is accompanied by a structural convergence to cross *β*-sheet conformation [1].

In Alzheimer’s disease (AD), a protein called amyloid-*β* (A*β*) peptide forms aggregates that deposit as senile plaques in brains. The nucleation-dependent process of A*β* aggregation is inferred by the occurrence of a ’lag-phase’ prior to fibril growth that shows a sigmoidal pattern [2]. In the aggregation pathway, one important intermediate called “protofibrils” has been isolated and characterized by several groups [3][4][5][6][7][8]. Protofibrils mainly differ from fibrils in their size and solubility; while fibrils can be sedimented with relatively smaller forces (19000g, 10min)-where *g* is the acceleration due to gravity- protofibrils require substantially high sedimentation forces and have smaller diameters than the fibrils [9]. Protofibrils have propensities to both elongate (by monomer addition) as well as to laterally associate (protofibril-protofibril association) to grow into mature fibrils and have been well characterized [9]. These two mechanisms of fibril growth depend on the structure and stability of protofibrils, which in turn depend on the factors affecting nucleation.

The *in vitro* A*β* aggregation process is well known to be affected by several environmental factors such as concentration, pH, ionic strength and temperature. In addition, differential effects of solvents on the aggregation process and size has also been documented [10]. Several biophysical methods are used to monitor the aggregation process among which, light scattering and centrifugation techniques are widely used to measure the hydrodynamic radii (a) as well as the diffusion coefficient (D) of the protein sample [5][11][12][13][14]. For spherical particles, the relation between *a* and *D* is given by the Stokes-Einstein (SE) equation;(1)

where, *k_B_* is Boltzmann’s constant, *T* is the absolute temperature and *η*_0_ is solution viscosity. However, the aggregates of A*β* peptide cannot be assumed to be spherical and hence determination of *a* can be erroneous. This difficulty is overcome by the use of multi-angle light scattering (MALS) technique which measures the intensity of the scattered light from multiple angles that in turn helps to calculate the molecular weight without assuming the shape of the solute [9]. A more sophisticated method of measuring the molecular size is by analytical ultracentrifugation (AUC), which utilizes the rate of the movement of sedimentation boundary [15]. For a spherical solute with a molecular mass, *MA* and radius *a* , the value of sedimentation coefficient measured at temperature *T* in buffer *b*, the rate of migration per unit field, *s_A_* is given by;(2)

where *N* is the Avogadro’s number and  is the partial specific volume of the solute, and *ρ*, *η* are the density and viscosity of the buffer medium respectively.

An important aspect of the Stokes-Einstein’s equation and its utility in protein aggregation systems in particular is that the viscosity of the bulk solvent in a given buffer solution is considered to be a constant.

**Figure 1:**
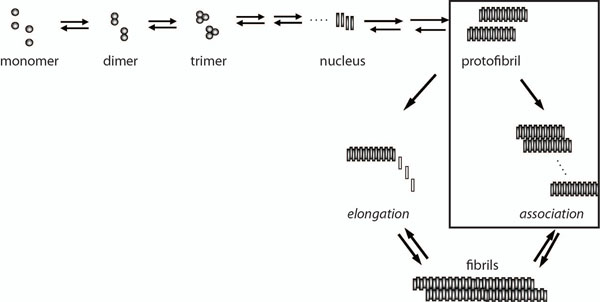
**Kinetics of A*β* aggregation pathway.** Schematic representation of the overall A*β* aggregation towards fibril formation indicating significant steps involved. The boxed area, which represents the conversion of protofibrils to fibrils via lateral association, is the reaction that is being investigated in the current work.

Here, we are reporting a modification in the classical Stokes-Einstein equation in order to accommodate the changes in viscosity of the solvent due to the changes in solvent size and shape, which is a more realistic model for A*β* aggregation. Specifically, we have focused on validating this model in protofibril lateral association reaction (Figure 1) along the aggregation pathway, which has been experimentally well characterized [9,16]. The modification we are reporting is not only useful in A*β* aggregation but also will be important for accurate measurement in all protein aggregation systems.

The classical SE model states that the diffusion coefficient for a suspension of molecules of a given volume fraction in a surrounding solvent of viscosity *η*_0_ is written as equation (1) where the denominator is the effective drag coefficient for the molecule which is treated as a rigid sphere of effective radius *a*. We point to several shortcomings of this model with respect to the A*β* aggregation and suggest modifications to improve it which include:

(i) firstly, molecules of concern to us are not necessarily spheres [17] although this is the simplest assumption one can make. In general for our problem, where we consider the aggregation of molecules, several classes of molecules (or n-mers) are assumed to populate the solvent. While it is not clear what the specific shape of each class (n-mer) is, it is most certainly not a sphere [18] for the most part.

(ii) The second point we make is that the SE relation takes the viscosity to be a constant, based upon the solvent viscosity. However we argue that it should depend upon the concentration of the various species of molecules present in the system. Any single molecule (individual or aggregated), during diffusion will experience the viscous forces of the liquid as well as due to the remaining molecules around it. As a result, the diffusion coefficient, *k*, is also a function of the concentration of the molecules. In fact, more generally, we will consider an effective diffusion coefficient *k_e_* where *k_e_* = *k_e_*(*ϕ_i_*,*η*_0_) where *i* = 1, 2, …,*n*.

In this paper, we have considered these two important conditions and incorporated modifications to the Stokes-Einstein equation that may faithfully represent the changes occurring in amyloid aggregation systems, especially A*β*. The significance of non-sphericity in computing the diffusion coefficient has been realized in various contexts and the biophysics, chemistry, geological and chemical engineering literature is replete with discussions of the role of non-spherical particles, specifically spheroidal particles [19-23]. While the biophysics community has been well aware of the importance of non-sphericity, the importance of concentration upon the viscosity and hence diffusivity, has been largely neglected. The chemical engineering literature is however a very rich source of discussion on this matter; see [24] and references cited therein for more information on this subject.

## Results

### Theoretical models

In a mixture or suspension containing a background liquid and *n*-species of molecules, we define *V*_0_ to be the volume of the solvent liquid and *V_i_*, *i* = 1, 2,…, *n* to be the volume of the *i-th* species. Then the volume fraction of each element of this mixture is given by:(3)

with the constraint:(4)

Based upon these arguments, a general expression for the diffusion coefficient for n-species of molecules can be framed in terms of the volume fraction *ϕ_i_* and must be of the form:(5)(6)

where *j* = 1, 2,…, *n*, *f_i_* refers to the drag coefficient of the *i-th* species and *β_i_*, its respective shape coefficient. As is seen from the last term on the right hand side of the equation, the volume fraction enters the diffusion coefficient through the viscosity. In fact, in this paper, we suggest that the viscosity term itself be treated as an average of the viscosity due to its constituents as a result of which *η_i_*(*ϕ_j_*) is replaced by a scalar, the effective viscosity, *η_e_*(*ϕ_j_*) where *j* = 1, 2,…, *n*. Hence:(7)

While we are following the vast majority of physics and engineering literature in following this approach of modeling a mixture through its overall material parameters, we are also conscious that it not a rigorous mathematical approach. In his paper on the “viscosities of mixtures” Massoudi [24] provides an overview of the historical approach to the modeling of effective viscosities but more importantly, he points to the mathematically rigorous approach of modeling a mixture by examining the stress tensors. However, this is recognized to be a rather formidable challenge to verify experimentally and very tedious to implement in computations such as these. Even within this approach of modeling viscosities, it has been argued [25] that the effective viscosity of say two liquids would be not just a linear superposition of the two individual viscosities, but also include an interaction term, referred to “mutual viscosity” which again is very difficult to verify experimentally. All these point to the complexity of rigorously modeling even a seemingly simple parameter as viscosity. In the rest of the paper, we consider two specific and successful models applied in the engineering literature to our problem of A*β* aggregation. With regards to the shape of the molecules, there has been much discussed about the validity of spherical-shape assumption of biomolecules( [17,18] and references therein) and while it is recognized that molecular shapes can be fairly complex and random, it still remains worthwhile to represent these arbitrarily composed molecules using some standard shapes that can be imagined to envelope the molecules in a reasonable manner. Our attempt in particular is to go beyond the assumption of the sphere. To do so we need at our disposal, the drag coefficients corresponding to arbitrarily shaped bodies. This is a very daunting challenge in fluid mechanics and at best relatively simpler shapes such as spheres, oblate and prolate spheroids (of any eccentricity) have been analyzed mathematically [26,27]. However there is some literature in terms of empirical or computational relations which provide the drag coefficients for other shapes.

### Case 1: a sphere-rod empirical model

We begin with an empirical model. Based upon the work of [28-31] we will consider the modification of the SE law by taking a suspension composing of spheres and rods with the former particles being considerably smaller than the latter such that the ratio *a_r_*/*a_s_* > 20, where *a_r_* is the major axis of the rod and *a_s_* is the radius of the sphere. This particular assumption can also be very meaningful in our case where we consider our sample primarily to be composed of smaller monomers and much larger protofibrils. In particular we follow the specific approach of [31] in obtaining an effective viscosity for such a suspension. In the words of [31] the primary assumption in the computation of viscosity is that:

... *for each fraction of a given particle size*, *the smaller particles in suspension have the same effect as a homogeneous fluid with Newtonian viscosity similar to the effective viscosity of a suspension made up of the fraction of smaller spheres. In other words*, *the smaller suspended particles do not interact with the larger particles and are ’sensed’ by the large particles as part of the continuous suspending fluid*.

Mathematically, this amounts to saying that:(8)

where *η*_*rel*,*r*_(*ϕ_r_*) refers to the viscosity of the rods relative to the continuum of a homogeneously distributed spheres in a solvent, , similarly is the viscosity of the spheres relative to the continuum of the pure solvent in the absence of the rods. Also, Φ_0_ = *ϕ_r_* + *ϕ_s_*, *ϕ_r_* refers to the concentration of the rods, *ϕ_s_* the concentration of spheres and:(9)

As in [31], based on the empirical relations suggested by [32,33] we take:(10)(11)(12)

Using these above equations along with the shape factor [27], where *L*, *b* refer to the length and diameter respectively, depending on the orientation of the rod, we have the relation for the diffusion coefficient, namely:(13)

for the case of *ϕ_r_* < 0.125. A similar relation can be written for *ϕ_r_* > 0.125 which is given by:(14)

If the net volume fraction of all the solute molecules is held fixed (i.e. Φ), then the diffusion coefficient can be written purely in terms of *ϕ_r_* or *ϕ_s_*.

### Case 2: a mixture theory approach

**Figure 2:**
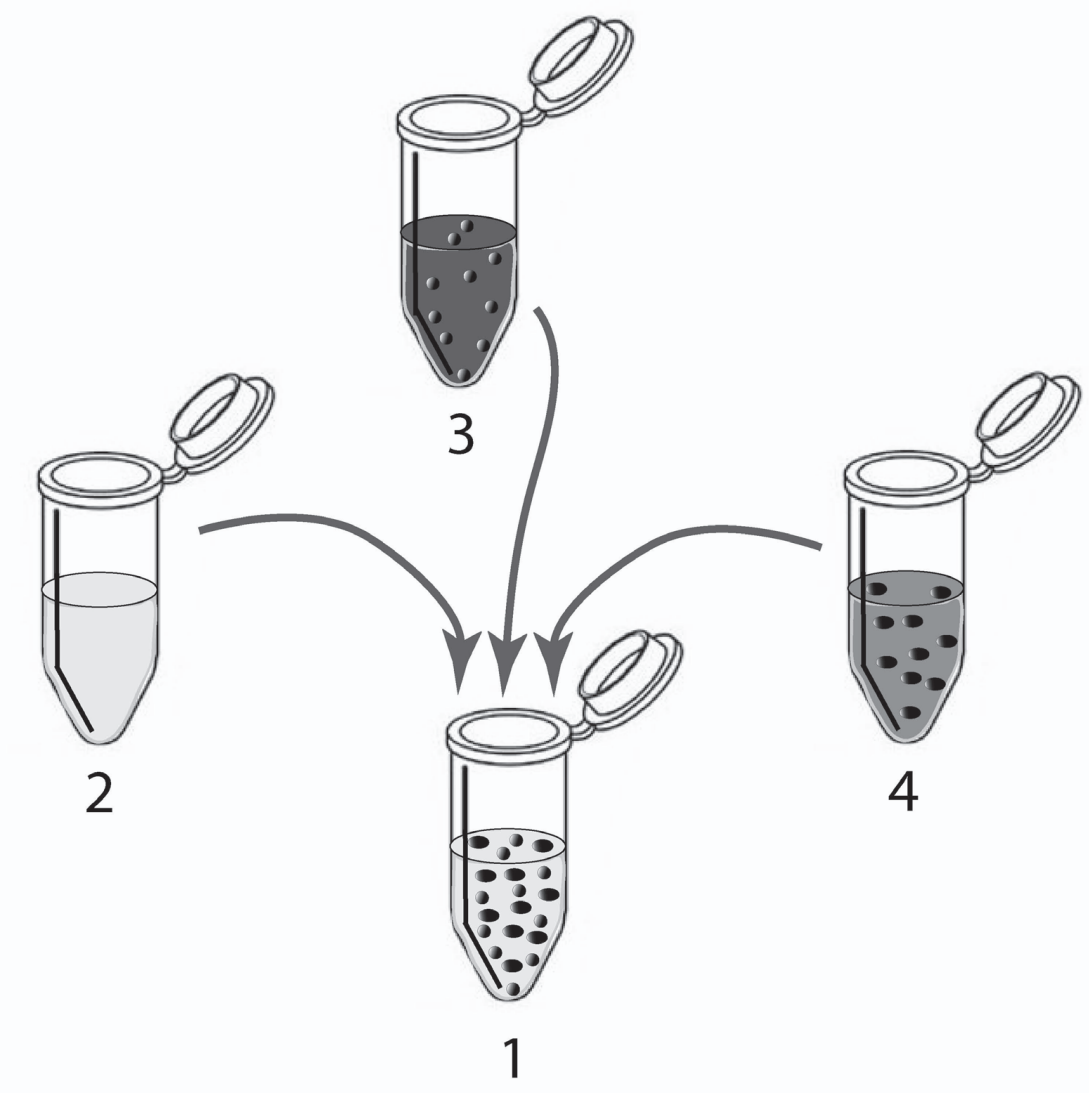
**Mixture theory based approach to find the effective viscosity.** A schematic showing the the mixture theory approach of estimating the effective viscosity of a 2-species suspension containing the pure solvent (L), species 1(S1) and species 2(S2) by means of a weighted average of the indivdual constituents of the suspension. The tube 2 contains the pure solvent L of viscosity *η*_0_; tube 3 contains S1 floating in a medium composed L and S2 and is of viscosity *η*_1_; tube 4 contains S2 in a medium composed of L and S1 which is of viscosity *η*_2_. By taking appropriate volumes from tubes 2,3 and 4, we prepare the suspension in tube 1 which now contains L, S1 and S2 which has an effective viscosity *η_e_* defined in equation (16).

In this section, we propose our model which is inspired by the empirical approach mentioned above and the viscosity model due to Quemada [34,35], who describes the “viscosity of suspensions of hard spheres or of structural units like clusters or aggregates” using the relation:(15)

where *η*_0_ is the viscosity of the background solvent, *ϕ* is the volume fraction of the suspensions and *ϕ_m_* is the maximum volume fraction at which the viscosity becomes singular, i.e. the critical limit of concentration beyond which flow ceases. The literature estimates 0.58 <*ϕ_m_* < 0.69 [36,37] (As in past literature, let us take the average value, namely *ϕ_m_* ≈ 0.6). In classical mixture theory the effective viscosity of two liquids of different viscosities has been taken to be the weighted average viscosity of the components. In attempting to do the same for suspensions, one must exercise a little caution. In the model proposed here, we adopt a mixture approach by trying to combine the solvent viscosity appropriately with equation (15). We schematically display our recipe for obtaining a suspension with solvent volume *V*_0_ and such that the volume of each constituent particle (molecule) is *V_i_* (*i* = 1, 2, *…*,*n*) in Figure 2, while requiring that equations (3) and (4) be satisfied.

Hence, based upon the definitions earlier, we define the effective viscosity as the weighted average of the individual components given by:(16)

where as in the sphere-rod model treated above, *η_i_*(*i* ≠ 0) where *i* ranges over all cyclic permutations of (1, *…*, *j*, *j* + 1, *…*,*n*), represents the relative viscosity of the suspension containing the *j*th solute in a “solvent” composed of *the liquid of viscosity η*_0_*and all other solute particles*. Note that the equation (16) is not unique. Other formulations of the effective viscosity are possible. The one provided here seems mathematically and physically reasonable and consistent with some models provided in the literature. The term  represents the relative volume fraction of the *j*’th solute relative to the “solvent” that contains it and is independent for different *j*’s. Therefore, for an n-species suspension, the viscosity relation for the *j* solute in a background containing all other species and the pure solvent can be inferred from the algorithm: *η_j_ → η_j_*_+1_*→* …*η_n_→ η*_1_*→* …*η_j_*_–1_ where *η_a_ → η_b_* indicates that *η_a_* is measured relative to *η_b_* in the sense of equation (8). In particular, the cyclic permutations of *i* are taken to ensure evenness in the definition of the volume fractions,  for all *j*’s. This is clearly illustrated in the cases of 2 and 3-species which are examined in detail. We illustrate this by means of a few examples.

#### (i) The case of two species

For instance, in the case of two solutes in a solvent, we can write the effective viscosity as:(17)(18)

where(19)

and similarly,(20)

where(21)

#### (ii) The case of three species

We can write the effective viscosity as:(22)(23)

where(24)

and similarly,(25)

where(26)

and also(27)

where(28)

We can similarly extend the computation of the effective viscosity to any number of solute species. Hence implementing this model into the above equation (7) for *k_e_* gives:(29)

where *η_e_* is defined by the equation (16) and  where *i*, *j* ≠ 0 that is, the fluid is discounted. Since the diffusion that we are interested in only pertains to the solute species embedded in the surrounding solvent, we need only consider the volume fraction of each solute with respect to the others in the evaluation of the effective diffusion. In particular let us assume, for instance, that two classes (or species) or molecules populate the solvent; the shape are so selected since the shape factors are only known for a small class of shapes. The first are the molecules at the protofibril stage or beyond which are treated as being spheroidal in shape(diffusion of spheroidal bodies had been considered as far back as 1936 by Perrin [38]) and the second class are those smaller than the protofibril stage which will be treated as spheres, even though Zwanzig and Harrison [18] rightly argue that the shapes of molecules are not correctly described by either of these shapes, the latter being far more realistic than the former. We however feel that this is still an improvement over the current approach of treating all the molecules as hard spheres.

**Figure 3:**
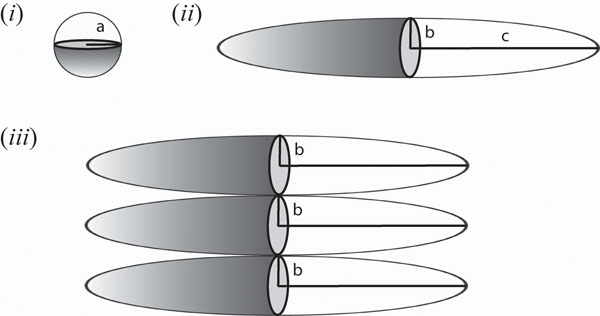
**Shapes of macromolecules and lateral association orientation.** The figures (i) and (ii) show the two different plausible shapes used to represent the protofibrils along with their appropriate dimensions. In the past these molecules have been represented as spheres for the most part. Based on experimental observations we suggest that a prolate spheroidal shape is more appropriate. Figure (iii) indicates the association mechanism of two spheroidal molecules. Since the ratio  , we can effectively assume that the association of two spheroids creates another spheroid with major and minor axes *c* and 2*b*, respectively.

If we define the total volume fraction of the molecules of both species to be Φ_0_ = 1, the volume fraction of the spheroidal particles to be *ψ*, then the volume of spherical molecules can be given by 1 – *ψ*. For the two species case we can write *ψ*_1_ = *ψ* and ψ_2_ = 1 – *ψ*. Therefore for this example, we can write the effective viscosity as:(30)

by taking a weighted average of the appropriate viscosities due to the three independent constituents. It must be noted that while the spherical shape is devoid of any orientational biases, the same cannot be said of the spheroid which can move about in different orientations  . The drag on a spheroid can be a maximum or minimum depending upon the orientation of the moving spheroid. We therefore estimate an upper and lower limit of the diffusion coefficient for the spheroidal molecules  where the lower and upper limits are obtained by the appropriate shape coefficients. These values corresponding to the spheroid and sphere (see Figure 3), respectively are known to be [26] (the chemistry and biophysics literature, in this regards, points to the model of Perrin [38]. We however use the more correct formula derived by Chwang and Wu [26] in 1975 using the Stokes singularity method):

Where  refers to the eccentricity of the spheroid *c* and *b* refer to the major and minor axis of the spheroid. Hence combining the above results allows us to formulate a specific model for the two-species case where the diffusion coefficient can be given by:(31)

while(32)

It is not difficult to see that in the limit that the spheroidal particles, vanish, i.e. the system contains the spherical molecules and solvent alone *ψ* = 1 and the effective diffusion coefficient can be written in the form:(33)

which is still an improvement over the classical SE model due to the accounting of the volume fraction in the viscosity. The classical formula is retrieved by ignoring the term  and taking *ϕ*_0_ + *ϕ* = 1 based on the constraint equation (4). For the one-species suspension case when *ϕ*/ϕ_0_ = ∈ << 1, then equation (33) can be approximately written at *O*(*∈*) as:(34)

where *ϕ*^(1)^ = *ϕ*. As ∈ *→* 0, the diffusion coefficient approaches the classical Stokes Einstein equation since then *ϕ*_0_*→* 1.

### Protofibril-protofibril lateral association experiment

**Figure 4:**
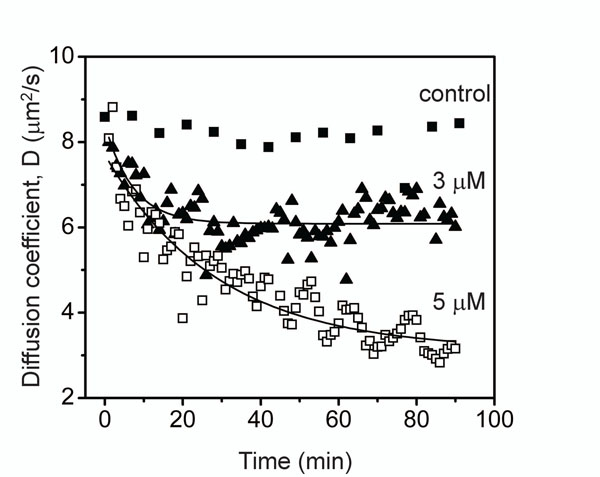
**Experimental esimation of diffusion coefficient versus time.** The lateral protofibrils association process monitored by dynamic light scattering (DLS). Isolated protofibrils (3 or 5 *µ*M) of A*β*42 buffered in 20 mM Tris-HCl, pH 8.0 was allowed to associate by the addition of 150 mM NaCl at room temperature. The reaction was monitored using DLS instrument for the shown period of time. The control sample was 5 *µ*M protofibril sample in the absence of NaCl.

Protofibril lateral association reaction was initiated by the addition of 150 mM NaCl to the isolated protofibrils (3 and 5 *µ*M) that was monitored by dynamic light scattering (DLS) instrument. Protofibrils without salt were used as a negative control. The data is plotted in Figure 4 against diffusion coefficient (D) which is inversely proportional to the hydrodynamic radius of the sample.

### Molecular level simulation of protofibril-protofibril lateral association

Next, we used some simple molecular level reaction models to predict the concentration of protofibrils of different size in the lateral association stage that can correspond to the diffusion coefficient estimates from DLS reported above. First, we assumed that the protofibrils are composed of 1600 monomers based on the previous reports [39,40]. Here, *F*_1600_ denote the protofibrils (comprising 1600-mers and average length 64 nm) [39,40]. In general, we will let *F_i_* denote a protofibril with *i* number of *Aβ* molecules binding to it during the lateral association phase.

We present separate reaction models considering different number of species formed during the lateral association reaction. The reactions involving these species were modeled under the assumption of a homogeneous mixture of protofibrils (with different initial concentrations) as used in the in vitro experiments. In particular, we considered a maximum of five different molecular species involved in this phase as follows: *F*_1600_, *F*_3200_, *F*_4800_ , *F*_6400_ and *F*_8000_. We have assumed that lateral association will result in at most a 5-fold increase in size of the protofibrils within the first 1.5 hrs of observation such that the system of reactions will not involve the formation of protofibrils beyond *F*_8000_. This assumption is purely theoretical although it is experimentally well known that three protofibrils associate together to form mature fibrils. We wanted to consider upto 5 species for the interest of modeling and calculations. The reaction fluxes in this are denoted by *R*(*i*, *j*) corresponding to the reaction between F_i__*1600_ and F_j__*1600_ forming *F*_(_*_i_*_+_*_j_*_)*1600_ for *i* = 1, …, 5 and *j* = *i*, *i* + 1,…, 5 – *i*. We have also assumed that the initial concentration of protofibrils comprise of only *F*_1600_’s and that of the other species are zero in the reaction models as only minute levels of associated protofibrils are expected to be present initially in the in vitro system.

In the following, we have considered three case studies to estimate the concentration change of the different molecular species involved in lateral association: 2-species (*F*_1600_, *F*_3200_), 3-species (*F*_1600_, *F*_3200_, *F*_4800_) and 5 species (*F*_1600_, *F*_3200_, *F*_4800_, *F*_6400_, *F*_8000_). Each reaction was considered reversible with *k*_+_ and *k*_–_ being the forward and backward rate constants respectively. Following our previously validated lateral association stage model [16], we have considered *k*_+_ = 9.0 × 10^–1^*h*^–1^*mM*^–1^ and *k*_–_ = 6.0 × 10^–3^*h*^–1^ respectively. While the 2-species and 3-species models are less realistic, we have seen that protofibrils do not grow beyond *F*_8000_ in the first 1.5 hrs of lateral association, and hence the 5-species model should be the best approximation of the in vitro system under study.

### Case 1: 2-species reaction model

### Case 2: 3-species reaction model

### Case 3: 5-speciesreaction model

After the kinetic schemes are established in the reaction models above, we computed the corresponding reaction fluxes, and hence the differential equations that govern the temporal change of these species can be derived from material balances and reaction kinetics. The reaction flux and differential equations for each of the models reported here have also been shown alongside. The initial concentration of protofibrils ([*F*_1600_]) is set to 1*µ*M, 3*µ*M and 5*µ*M respectively following the in vitro experiments while the concentrations of the other species are assumed to be zero at the start (i.e., [F_i__*1600_] = 0, *i* = 2,…, 5). Thus, the system of differential equations for each model is properly defined and were solved using Matlab’s ODE toolbox to estimate the concentration of the different species at various time points between 0 – 90 mins as shown in the previous section.

## Case studies

In this section, we consider some special cases to test our proposed models. We consider the cases of two, three and five species embedded in a solvent, introduced earlier. In these examples, we consider each species to be an ellipsoid since the length and width of the molecules considered here are significantly different so they cannot be correctly represented as spheres. As is indicated in Figure 3(c), associations of these spheroids occur laterally; therefore according to our model, the result of n-associations of spheroids of major axis c and minor axis *b* results in a new spheroid with major axis c and minor axis *n* × *b*. Our objective is to employ our model to estimate the effective diffusion coefficient of various systems and compare them to the experimentally measured values of diffusion.

**Figure 5:**
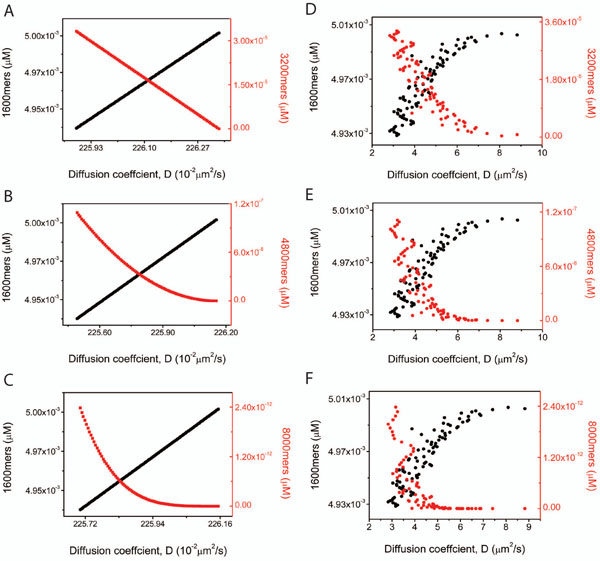
**Experimental and theoretical comparison of the diffusion coefficient.** This figure shows the variation in diffusion coefficient of 1600-, 3200-, 4800- and 8000-mers corresponding to their concentrations for the three case studies of 2-(row 1), 3-(row 2) and 5-species(row 3). The panels on right show the experimental results while those on the left correspond to the theroretical predictions. Each panel shows two curves; the black curves represent the diffusion coefficients of the smallest molecules (1600-mers in each case) while the red curves represent the diffusion coefficients of the largest molecules namely: 3200-mers, 4800-mers and 8000-mers respectively corresponding to the 2-,3- and 5-species cases.

For our calculations, we take the dimensions of the protofibril (1600-mer) to be a spheroid with minor axis equal to 2.25nm and the major axis to be 300nm based upon previously published reports [8,9,41]. Also we take the viscosity of the solvent (water) to be 0.89 × 10^–3^ Pa-s. The results of our computations are plotted in Figure 5. The right columns of this plot (Figures 5D, E and F) show the results of the experiments which yield the diffusion coefficient and the concentrations alike. The initial concentration of the protofibril is taken to be 5*µM* in each of the cases. The black points in the curves refer to the 1600 mers while the red points refer to the final stage in each case, i.e. 3200mers in the 2-species case, 4800 mers in the 3 species and the 8000 mers in the 5 species case. Note that the diffusion coefficient always decreases with the diminishing 1600 mers on the other hand growing with the diminishing concentrations of the bigger, associated molecule. The panels on the left hand side (Figures 5A, B and C) indicate the results of the numerical computations for the same cases as the experiments and indicate very similar trends. For ease of comprehension of the plots the values of  alone are shown in the graphs here while those of  show very similar features. It is to be noted that the black and red points do not actually intersect; the intersection is an artefact of the two different scales represented on the y-axis. While the increasing and decreasing trends remain consistent with observations the actual value of the diffusion coefficient seems to differ. Our model puts the lower and upper limit values of the diffusion coefficient consistently between 1.7 × 10^–12^*m*^2^/s and about 2.4 × 10^–12^*m*^2^/*s* while experimental values are higher, ranging upto four times more that our upper limit at some concentrations. Firstly the large variability in the experimental data when compared to our model can be attributed to the fact that the experimental study is based upon DLS observations which provides a time varying effective radius for the system, corresponding to the production of larger molecules which form as a result of the association. These experimentally evaluated radii which range between 30-80nm are then inserted into the classical Stokes-Einstein formula (equation (1)) to get the diffusion coefficient. The experimental estimates for the effective length scale are substantially lower than the more accurate values used in our model, resulting in the deviation from our values of the diffusion coefficient.

Certain features of the computations merit immediate attention. In particular, the profile of the 1600, 3200 and 8000mers is striking. These include the declining values of the apparent intersection point with increasing number of species and also the slope of the red curves whose declining trend in the graph as plotted, gets more rapid with the larger molecule.

**Figure 6:**
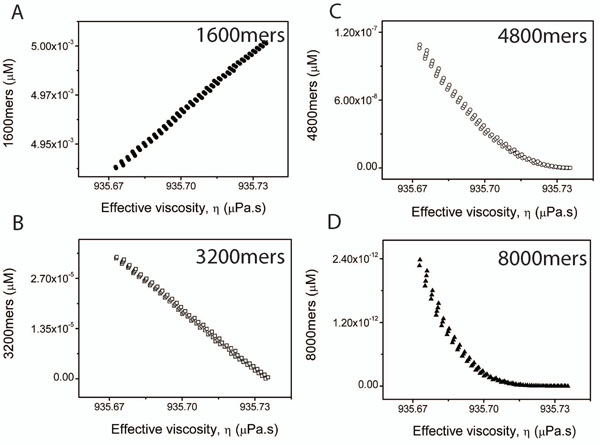
**Variation of effective viscosity with concentration.** Figures A,B,C and D show variations in the effective viscosity versus concentration in the 5-species example for 1600-mers, 3200-mers, 4800-mers and 8000-mers respectively. The figure A shows that the effective viscosity is directly proportional to the concentration of the 1600-mers while B,C and D reveal it to be inversely proportional to the respective concentrations.

The most significant contribution of the paper lies in the suggestion that the viscosity of the system is not a constant as considered so far but depends strongly upon the concentration of the various molecules. In Figure 6, we plot the change in the effective viscosity of the 2-, 3- and 5-species cases as a function of the concentrations of the 1600, 3200, 4800 and 8000 mers. A clear declining trend is observed which is in agreement with the literature [42-44]. Viscosity of suspensions have been known to diminish with the declining concentrations of the solute. On the other hand viscosity increases with the decreasing size of the solute particles since smaller particles are capable of more random Brownian motions causing greater retardation of the bulk fluid motion. The approach proposed in this paper must not be thought simply as a reaction to the DLS estimates which essentially uses the equation (1). As one can note from equation (2), even the AUC method relies upon the drag formula corresponding to a sphere and the viscosity of the buffer does not depend upon the volume fraction of the embedded solutes.

## Discussion and future work

The intention of this paper is to point out a somewhat erroneous practice of employing the Stokes-Einstein (SE) formula in situations which are not represented by it accurately enough. The proposed modification is a first in a series of steps that will help provide better mathematical models that fully incorporate all features of the entire system to explain protein aggregation. While the shape factor and concentration are introduced as tuning parameters, the central contribution of this paper lies in the realization that the meaning of “viscosity”(denoted *η*_0_) in the Stokes-Einstein formula is the one pertaining to the solvent alone. However if one wants to think of the diffusion of any molecule, the effective medium becomes the solvent as well as the remaining molecules embedded in the system. Therefore the solvent viscosity of the classical SE formula needs to be replaced appropriately with one of an effective viscosity which is no longer just the solvent viscosity alone. In fact the concentration terms are as important if not more than the non-sphericity requirement. It is also important to note that while the apparent form of the modified SE formula is similar to that of the classical SE formula, our model has grave implications in understanding the dynamics of the protofibrils and aggregate molecules since the diffusion coefficient is no longer a constant but a function of the shape factors and concentrations of the different species of molecules that populate the suspension. While the change from a sphere to a spheroid might appear to be a minor point, its full impact can only be realized when one studies the problem at the smaller scale where this change can display its full impact as we plan to do in the future; this paper is focused on the larger scale continuum properties of the system as experimental measurements were hard to achieve at a smaller scale for the protein aggregation problem. To see the complexity of this model, one need only look at the diffusion equation that our model generates. For instance, in the case of n-species of n-mers embedded in a solvent,(35)

where *i* = 1,2,…,*n*. The new *k_e_* renders the equation highly nonlinear. The modified SE equation provided in this paper now allows for a more appropriate coupling of the solute-solvent dynamics. Under the realistic conditions of flow we have a two way coupling; the dynamics of the solute is described by the concentration equation (35), while the dynamics of the solvent would be given by the Navier-Stokes equation. Our next goal is to take on this multiscale approach coupled with a reaction term to understand the temporal and spatial variations in the solute (protofibrils and aggregates) distribution. Due to the contributions of this paper, this would be a very new and rigorous addition to the biophysics literature. The model proposed here, therefore merits serious attention since it provides a systemic treatment of the biophysics involved. The current paper is therefore a first step in this long term approach of understanding the problem of protein aggregation thoroughly and rigorously.

## Conclusion

In this report, we have shown that the use of classical Stokes-Einstein equation may not be accurate for the measurement of hydrodynamic radii and consequently molecular weights of amyloid aggregates. Although it is known that appropriate assumption of the shape of solute molecules is essential for accurate size determination while using Stokes-Einstein equation, this is the first time we have shown that it is incorrect to assume solvent viscosity be a constant during the reaction. This is particularly true for reactions involving A*β* protofibrils, as large size of the solute molecules indicate significant effect on bulk solvent viscosity. We have attempted to rectify the problem by incorporating modifications to the Stokes-Einstein equation (Equation 16)that seem to be valid for the protofibril lateral association reaction. It is noteworthy that the modifications reflecting the viscosity changes can lead to realistic models not only for A*β* aggregation but also for a majority of amyloid aggregation systems.

## Methods

### A*β* protofibril preparation

Synthetic A*β*42 peptide was obtained from synthesis facility at Mayo Clinic, Rochester, MN as a lyophilized powder. A*β*42 protofibrils were generated and isolated as previously reported (9). Freshly purified A*β*42 monomers (100 *µ*M) from size exclusion chromatography buffered in 10 mM Tris, 50 mM NaCl, pH 8.0 was agitated at room temperature for 48 h. The aggregation was monitored using ThT fluorescence. The sample was then centrifuged at 19000g for 12 min to spin out any fibril that may have formed. The supernatant was then fractionated by Superdex-75 size exclusion column to isolate protofibrils from unreacted monomers and smaller oligomers. The concentration of protofibrils was measured by UV-Vis with a molar extinction coefficient of 1450 *cm*^–1^*M*^–1^ corresponding to A*β*42.

### Dynamic light scattering (DLS)

The DLS experiments were performed using Zetasizer nanoseries instrument ( Malvern Instruments, Inc.). The samples were equilibrated for 120s and a total of 90 measurements of 10s each were made with a time interval of 1min after each measurement. The data was exported as diffusion coefficient using Zetasizer software version 6.20 and processed using origin 6.0 software.

### Reaction flux and differential equations for the reaction pathway

After the kinetic schemes for each of the three reaction models are established, the concentrations of the various species can be expressed as functions of time by using material balances and reaction kinetics. We will explain the 5-species reaction model in this section, while the other two models can be derived similarly. The reaction fluxes for the 5-species model can be derived as follows:

Next, we formulate the differential equations for each species in the system to model their change in concentration with time. The first species to be considered is *F*_1600_ whose rate of change is expressed in terms of its disappearance:

(and hence the negative sign before each reaction flux term) due to each of the reactions involving *F*_1600_ enlisted in 5-species reaction model. Similarly, the differential equations of each of the other species involved can be written based on the fluxes of the reactions where the species is produced (positive terms) or consumed (negative terms):

The initial concentration of *F*_1600_s is equal to the amount of protofibrils added initially and is the main driving force for the downstream reactions. The concentrations of the other species are assumed to be zero at the start (i.e., [*F_i_*_×1600_] = 0, *i* = 1, …, 5). This set of differential equations is properly defined and can be solved using Matlab’s ode15s.

## Competing interests

The authors declare that they have no competing interests.

## Authors contributions

The study was conceptualized by PG,VR and AV. PG designed the computational model for the reaction pathway and VG designed the ThT fluorescence and DLS experiments. AV,SA and BJC performed the mathematical modeling and the related calculations. All authors read and approved the final manuscript.

## References

[B1] Tycko R Molecular structure of amyloid fibrils: insights from solid-state NMR Q Rev Biophys 2006 1 55 10.1017/S0033583506004173 16772049

[B2] Harper JD Lansbury PT Jr. Models of amyloid seeding in Alzheimer’s disease and scrapie: mechanistic truths and physiological consequences of the time-dependent solubility of amyloid proteins Annu Rev Biochem 1997 66 385 407 10.1146/annurev.biochem.66.1.385 9242912

[B3] Walsh DM Lomakin A Benedek GB Condron MM Teplow DB Amyloid beta-protein fibrillogenesis. Detection of a protofibrillar intermediate J. Biol. Chem 1997 272 22364 22372 10.1074/jbc.272.35.22364 9268388

[B4] Ward RV Jennings KH Jepras R Neville W Owen DE Hawkins J Christie G Davis JB George A Karran EH Howlett DR Fractionation and characterization of oligomeric, protofibrillar and fibrillar forms of beta-amyloid peptide Biochem. J 2000 348 1 137 144 10.1042/0264-6021:3480137 10794724 PMC1221046

[B5] Murphy RM Pallitto MM Probing the kinetics of beta-amyloid self-association J Struct Biol 2000 130 109 122 10.1006/jsbi.2000.4253 10940219

[B6] Harper JD Wong SS Lieber CM Lansbury PT Jr. Assembly of A beta amyloid protofibrils: an in vitro model for a possible early event in Alzheimer’s disease Biochemistry 1999 38 8972 8980 10.1021/bi9904149 10413470

[B7] Blackley HK Sanders GH Davies MC Roberts CJ Tendler SJ Wilkinson MJ In-situ atomic force microscopy study of beta-amyloid fibrillization J. Mol. Biol 2000 298 833 840 10.1006/jmbi.2000.3711 10801352

[B8] Harper JD Wong SS Lieber CM Lansbury PT Observation of metastable Abeta amyloid protofibrils by atomic force microscopy Chem. Biol 1997 4 119 125 10.1016/S1074-5521(97)90255-6 9190286

[B9] Nichols MR Moss MA Reed DK Lin WL Mukhopadhyay R Hoh JH Rosenberry TL Growth of beta-amyloid(1-40) protofibrils by monomer elongation and lateral association. Characterization of distinct products by light scattering and atomic force microscopy Biochemistry 2002 41 6115 6127 10.1021/bi015985r 11994007

[B10] Shen CL Murphy RM Solvent effects on self-assembly of beta-amyloid peptide Biophys. J 1995 69 640 651 10.1016/S0006-3495(95)79940-4 8527678 PMC1236289

[B11] Bitan G Kirkitadze MD Lomakin A Vollers SS Benedek GB Teplow DB Amyloid beta -protein (Abeta) assembly: Abeta 40 and Abeta 42 oligomerize through distinct pathways Proc. Natl. Acad. Sci 2003 100 U.S.A 330 335 10.1073/pnas.222681699 12506200 PMC140968

[B12] Pallitto MM Murphy RM A mathematical model of the kinetics of beta-amyloid fibril growth from the denatured state Biophys. J 2001 81 1805 1822 10.1016/S0006-3495(01)75831-6 11509390 PMC1301655

[B13] Hepler RW Grimm KM Nahas DD Breese R Dodson EC Acton P Keller PM Yeager M Wang H Shughrue P Kinney G Joyce JG Solution state characterization of amyloid beta-derived diffusible ligands Biochemistry 2006 45 15157 15167 10.1021/bi061850f 17176037

[B14] Smith AM Jahn TR Ashcroft AE Radford SE Direct observation of oligomeric species formed in the early stages of amyloid fibril formation using electrospray ionisation mass spectrometry J. Mol. Biol 2006 364 9 19 10.1016/j.jmb.2006.08.081 17005201 PMC7618241

[B15] Schuck P Perugini MA Gonzales NR Howlett GJ Schubert D Size-distribution analysis of proteins by analytical ultracentrifugation: strategies and application to model systems Biophys. J 2002 82 1096 1111 10.1016/S0006-3495(02)75469-6 11806949 PMC1301916

[B16] Ghosh P Kumar A Datta B Rangachari V Dynamics of protofibril elongation and association involved in Abeta42 peptide aggregation in Alzheimer’s disease BMC Bioinformatics 2010 11 Suppl 6 S24 10.1186/1471-2105-11-S6-S24 20946608 PMC3724481

[B17] Cantor CR Shimmel PR Biophysical chemistry: Part II: Techniques for the study of biological structure and function 1980 W.H. Freeman

[B18] Zwanzig R Harrison AK Modifications of the Stokes-Einstein formula Journal of Chemical Physics 1985 83 11 5861 5863 10.1063/1.449616

[B19] Gregorova E Pabst W Bouchet JB Influence of particle shape on the viscosity of Kaolin suspensions Acta Geodyn. Geomater 2009 6 1 101 109

[B20] Shenhar R Wang H Hoffman RE Frish L Avram L Willner I Rajca A Rabinovitz M Self-Assembled, helically stacked anionic aggregates of 2,5,8,11 tetra tert butylcycloocta [1,2,3,4-def;5,6,7,8-def]bisbiphenylene, stabilized by electrostatic interactions J. Am. Chem. Soc 2002 124 4685 4692 10.1021/ja012140u 11971717

[B21] Vasanthi R Ravichandran S Bagchi B Needlelike motion of prolate ellipsoids in the sea of spheres J. Chem. Phys 2001 114 7989 10.1063/1.1363674

[B22] Stein A Hoffmann DA Marcus AH Leezenberg PB Frank CW Fayer MD Dynamics in poly(dimethylsi1oxane) melts: fluorescence depolarization measurements of probe chromophore orientational relaxation J. Phys. Chem 1992 96 5255 5263 10.1021/j100192a017

[B23] Kim MS Study of molecular reorientation in liquid with Raman spectroscopy (III). Temperature dependence of molecular rotation of C6Fc in neat liquid J. of Korean Chem. Soc 1984 28 1 34 40

[B24] Massoudi M A note on the meaning of mixture viscosity using the classical continuum theories of mixtures International Journal of Engineering Science 2008 46 677 689 10.1016/j.ijengsci.2008.01.008

[B25] Sampaio R Williams WO Viscosities of liquid-mixtures, Zeitschrift Angew Math. Phys 1977 28 ZAMP 607 614

[B26] Chwang AT Wu TY Hydromechanics of low-reynolds-number flow. Part 2. Singularity method for Stokes flows Journal Fluid Mechanics 1972 45 105 145

[B27] Happel V Brenner H Low Reynolds number hydrodynamics 1965 Prentice Hall

[B28] Chang C Powell RL Dynamic simulation of bimodal suspensions of hydrodynamically interacting spherical particles Journal of Fluid Mechanics 1993 253 1 25 10.1017/S0022112093001697

[B29] Farris RI Prediction of the viscosity of multimodal suspensions from unimodal viscosity data Journal of Rheology 1968 12 281 10.1122/1.549109

[B30] Fidleris V Whitmore RL The physical interaction of spherical particles in suspensions Rheologica Acta 1961 I 573

[B31] Morr R Gottlieb M Graham A Mondy L Viscosity of concentrated suspensions of sphere/rod mixtures Chemical Engineering Communications 1996 148 1 421 430

[B32] Milliken WJ Mondy LA Gottlieb M Graham AL Powell RL The effect of the diameter of falling balls on the apparent viscosity of suspensions of spheres and rods Phys. Chem. Hydro 1989 11 341

[B33] Thomas DG Transport characteristics of suspension: VIII. A note on the viscosity of Newtonian suspensions of uniform spherical particles Journal of Colloid Science 1965 20 267 10.1016/0095-8522(65)90016-4

[B34] Quemada D Rheology of concentrated disperse systems. I. Minimum energy dissipation principle and viscosity-concentration relationship Rheologica Acta 1977 16 1 82 94 10.1007/BF01516932

[B35] Quemada D Rheological modelling of complex fuids. I. The concept of effective volume fraction revisited European Physical Journal of Applied Physics 1998 1 1 119 127 10.1051/epjap:1998125

[B36] Lee JD So JH Yang SM Rheological behavior and stability of concentrated silica suspensions Journal of Rheology 1999 43 5 1117 1140 10.1122/1.551018

[B37] Wolthers W Van den Ende D Duits MHG Mellema J The viscosity and sedimentation of aggregating colloidal dispersions in a Couette flow Journal of Rheology 1996 40 1 55 67 10.1122/1.550784

[B38] Perrin F Brownian movement of an ellipsoid J. Phys. Radium 1936 7 1 11 10.1051/jphysrad:01936007010100

[B39] Blackley HG Sanders GH Davies MC Roberts CJ Tendler Sj Wilkinson MJ In-situ atomic force microscopy study of beta-amyloid fibrillization J Mol Biol 2000 298 833 840 10.1006/jmbi.2000.3711 10801352

[B40] Nichols MR Moss MA Reed Dk Lin WL Mukhopadhyay R Hoh JH Rosenberry TL Growth of beta-amyloid(1-40) protofibrils by monomer elongation and lateral association. Characterization of distinct products by light scattering and atomic force microscopy Biochemistry 2002 41 6115 6127 10.1021/bi015985r 11994007

[B41] Goldbury C Frey P Olivieri V Aebi U Muller S Multiple assembly pathways underlie amyloid-beta fibrill polymorphisms J. Mol. Biol 2005 352 292 298 10.1016/j.jmb.2005.07.029 16095615

[B42] Barthelmesa SE G. Pratsinisa H Buggisch B Particle size distributions and viscosity of suspensions undergoing shear-induced coagulation and fragmentation Chemical Engineering Science 2003 58 2893 2902 10.1016/S0009-2509(03)00133-7

[B43] Naoya H Ikuro S Experiments on granular rheology: Effects of particle size and fluid viscosity Journal of Geophysical Research 2009 114 B04413

[B44] Phan-Thien N Pham DC Differential multiphase models for polydispersed pheroidal inclusions: thermal conductivity and effective viscosity International Journal of Engineering Science 2008 38 73 88

